# Basketball detection based on YOLOv8

**DOI:** 10.1371/journal.pone.0326964

**Published:** 2025-08-26

**Authors:** Zeyu Liang, Jiuyuan Wang, Tianhao Huang, Zilong Sang, Jia Zhang

**Affiliations:** 1 School of Chinese Basketball, Beijing Sport University, Beijing, China; 2 JTSportech Research Institute of Sport Sciences, Beijing, China; 3 School of Exercise and Health Sciences, Beijing Sport University, Beijing, China; 4 College of Physical Education, Chongqing University, Chongqing, China; University of Hong Kong, HONG KONG

## Abstract

Accurate and timely detection of basketballs is crucial for ensuring fairness in games, enhancing the precision of data analysis, optimizing tactical planning for coaches, and improving the spectator experience. However, current basketball detection technologies face challenges such as variations in target scale, scene complexity, and changing camera angles, which limit automated systems’ accuracy and real-time performance. To address these issues, this study introduces a novel real-time basketball detection model, BGS-YOLO, incorporating several key innovations. First, the model integrates a BiFPN (Bidirectional Feature Pyramid Network) that enhances detection accuracy by efficiently merging feature maps across different resolutions, allowing for more effective feature extraction from basketball targets. Second, the Global Attention Mechanism (GAM) dynamically adjusts the model’s focus, optimizing feature attention in complex or partially occluded scenes, boosting recall in occluded scenarios by 3.2%, thereby improving localization precision. Finally, SimAM-C2f increases the model’s robustness in high-interference environments by calculating similarity features between the target and the background, reducing false positives by 15%, ensuring more reliable detection. Experimental results show that BGS-YOLO surpasses existing models across key metrics such as precision, recall, F1 score, and mean average precision (mAP), achieving a mAP of 93.2%. All improvements were statistically significant (p < 0.001). These advancements significantly enhance the accuracy and robustness of basketball detection, offering valuable technical support for intelligent sports analytics.

## Introduction

Basketball is a globally popular sport and plays a crucial role for athletes, coaches, and spectators, particularly in ensuring game fairness and data accuracy [1–3]^.^ Automated basketball detection systems improve game fairness and entertainment [4]. However, current technologies face challenges in accuracy, real-time performance, and adaptability to complex environments [5,6]. Therefore, developing an advanced basketball detection method could not only increase automation in games but also provide robust technical support for data analysis, training assistance, and live broadcasting [7,8].

Historically, research on basketball detection has been divided into traditional methods and computer vision-based approaches. Traditional methods include manual observation, recording, and basic sensor usage [9]. Early studies relied primarily on referees’ visual observation to assess events in basketball games, such as scoring and fouls [10]. The reliance on human vision has been widely debated, with research focusing on its accuracy and consistency [11]. In small-scale or amateur games, manual scoring and statistical recording remain common practices. However, this approach is prone to human error and struggles to keep pace with the speed of basketball games [12]. Some studies have investigated the use of basic sensors, such as infrared or pressure sensors, to automatically record scoring events in basketball games. While these methods have improved automation to some extent, their detection range and accuracy remain limited [13,14].

Research in computer vision includes both traditional machine learning and deep learning techniques [15]. Recent works like sparse feature selection via hypergraph Laplacian and robust semi-supervised multi-label learning have advanced feature engineering in sports analytics. Machine learning, a branch of artificial intelligence, enables systems to learn from data and make decisions or predictions without explicit programming [16]. For basketball detection specifically, BiFPN was adopted over conventional FPN due to its dynamic weight normalization capability, which is particularly effective for handling the wide range of basketball sizes resulting from varying camera distances and angles during gameplay. Traditional machine learning methods include decision trees, support vector machines (SVM), and naive Bayes, among others [17–19]. Georgios K et al. [20] improved 18 key performance indicators in basketball using decision tree algorithms, achieving a weighted absolute percentage error (WAPE) of 34.14%. Zhao et al. [21] utilized support vector machines (SVM) to classify four basketball postures, demonstrating that after principal component analysis (PCA) dimensionality reduction, the accuracy of SVM inputs notably increased. Ball et al. [19] evaluated European basketball leagues with a mixed four-factor DefenseOffense model, exploring logistic regression, naive Bayes, and other techniques, ultimately achieving a 98.90% success rate with the dataset model. Huang et al. proposed a multimodal predictive model, exhibiting superior performance compared to submodels with an average accuracy increase of 8.2% and 20.3%. However, these methods are limited by their reliance on manually designed features, requiring extensive feature engineering, which is time-consuming and prone to subjective biases. This can result in the omission of crucial features or an overemphasis on suboptimal ones, ultimately limiting model performance [22,23].

Deep learning methodologies are primarily categorized into two-stage deep learning methods and one-stage deep learning methods [24]. Two-stage deep learning methods represent a form of target detection technology involving two core stages: region proposal and precise detection. In the initial stage, the system generates candidate regions potentially containing target objects, while in the subsequent stage, these regions are classified, and the boundary boxes are finely adjusted. Two-stage deep learning algorithms primarily encompass the R-CNN series algorithms, including the original R-CNN, Fast R-CNN, Faster R-CNN, and Mask R-CNN, among others [25,26]. Roche et al. [27] introduced a framework leveraging multimodal machine learning advantages to address activity recognition challenges. Chen et al. [28] presented a two-stage deep learning approach for rapid and accurate landmark detection. Xu et al. compared Faster R-CNN and Mask R-CNN, highlighting the effectiveness of joint training strategies. However, the escalated computational complexity and processing time constrain the practicality of two-stage deep learning methodologies in real-time or near-real-time scenarios [29]. Consequently, single-stage deep learning methods have progressively emerged as the preferred option in basketball detection research due to their reduced computational costs and resource requirements [30].

One-stage deep learning methods have garnered attention for their efficient target detection capabilities. Notably, the YOLO (You Only Look Once) series is distinguished for its rapid direct prediction, SSD (Single Shot MultiBox Detector) for its multi-scale detection capability, and RetinaNet for its multi-scale feature capturing and focal loss optimization. Wang et al. [31] proposed a novel deep-learning mechanism for identifying athletes’ foul behaviors. Shi et al. [32] introduced a new deep-learning approach inspired by SSD to tackle localization issues. Peng et al. [33] presented the WeedDet algorithm based on RetinaNet, significantly enhancing performance metrics. Wang et al. [34] introduced an enhanced YOLO V4 method that delivered impressive outcomes on the DOTA dataset. Liu et al. [35] proposed an improved object detection algorithm based on enhanced YOLOv5 for basketball applications. YOLOv7 further refined speed and accuracy through faster convolution operations and smaller models.

Furthermore, YOLOv7 leverages quicker convolution operations and smaller models, enhancing speed and accuracy. Zhang et al. [36] developed the YOLOv7-RDD model for road detection, showcasing superior efficiency. Wu et al. [37] devised the enhanced object detection network YOLO-RGBDtea based on YOLOv7, demonstrating enhanced performance in intricate outdoor settings. Liu et al. [38] introduced an algorithm based on YOLOv8 design, boosting the feature extraction capabilities of the backbone network. Experimental results exhibit that this algorithm achieves a mAP of 79.9%. Nevertheless, YOLOv8 encounters challenges in detecting small objects, extracting features in complex scenes, and generalization capabilities. The substantial computational resource demands may also restrict its applicability in resource-constrained environments [39].

Building on previous research, both traditional machine learning and deep learning approaches have advanced basketball detection, yet significant limitations persist. Traditional machine learning methods depend on manually crafted features, which are not only time-consuming and labor-intensive but also subject to human bias. This can result in the omission of critical features or the overemphasis of suboptimal ones, thereby constraining model performance. In contrast, deep learning techniques, which autonomously learn features, have gained widespread application in object detection. However, existing deep learning models, such as two-stage R-CNN algorithms, although highly accurate, suffer from high computational complexity and long processing times, limiting their feasibility for real-time or near-real-time applications. Single-stage models, such as the YOLO series, offer superior speed and computational efficiency yet continue to face challenges in detecting small objects and extracting features in complex scenes.

To overcome these limitations, this study proposes the BGS-YOLO model. Through a series of ablation experiments, the effectiveness of its components was validated and optimized. By sequentially incorporating the Bi-directional Feature Pyramid Network (BiFPN), Global Attention Mechanism (GAM), and Similarity Attention Mechanism (SimAM-C2f), the model’s feature extraction capabilities for basketball targets were significantly enhanced, resulting in marked improvements in detection accuracy and robustness in complex environments. The BiFPN efficiently integrates multi-scale features, facilitating better object detection across various target sizes. GAM optimizes feature selection by concentrating on information-rich regions of the image, while SimAM-C2f enhances the model’s ability to distinguish between targets and backgrounds by computing similar features. The synergy of these components enables the BGS-YOLO model to achieve high accuracy and robustness, particularly in managing complex scenes and dynamic conditions in basketball detection tasks.

## Materials and methods

### YOLOv8 model

**Fig 1:**
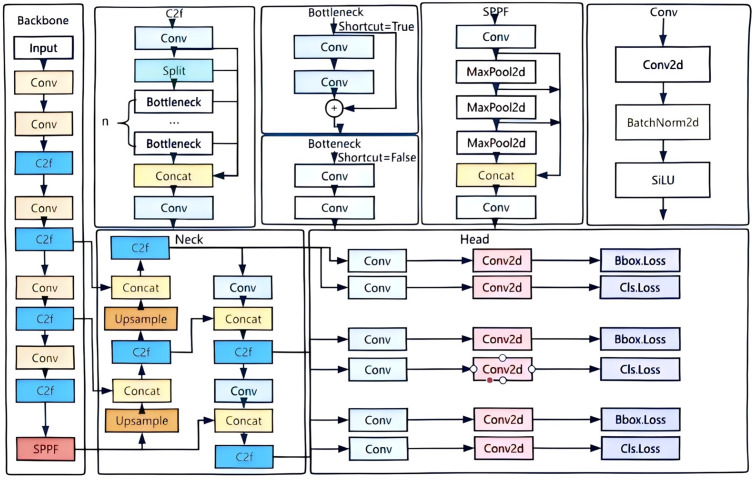
Structure of YOLOv8n.

YOLOv8 represents the latest advancement in the field of object detection, inheriting and refining the core principles of the YOLO series. The YOLOv8 model series includes several variants of different scales and performance characteristics, denoted by the letters “x,” “n,” “l,” and “m,” with the “n” version specifically designed to balance speed and accuracy. The “n” version of YOLOv8 consists of several key components, including the input layer, backbone layer, neck, and output layer. These components work together to ensure that the model delivers accurate object detection results while maintaining computational efficiency [40–42]. The structure of the YOLOv8n algorithm is illustrated in Fig 1.

First, the input layer is responsible for preprocessing the raw image data, laying the foundation for subsequent feature extraction. This layer typically involves operations such as image normalization and resizing to ensure that the input data meets the model’s requirements and can be efficiently processed within the network. By converting the image data through the input layer, it is optimized into a format suitable for deep learning networks, thereby enhancing the computational efficiency and detection accuracy of the subsequent layers [43–45].

The backbone layer is the core component of the model, responsible for extracting rich feature information from the input image through a series of convolution and downsampling operations. The backbone layer of YOLOv8 builds upon the classic structure of the YOLO series, with optimizations aimed at enhancing feature extraction capabilities and computational speed. This layer’s design not only maximizes information extraction efficiency but also ensures the model’s outstanding performance across various tasks [46–48].

The neck layer, serving as a crucial bridge between the backbone and output layers, is responsible for further integrating and processing feature information. In YOLOv8, the neck layer utilizes a specific feature pyramid structure that allows for the fusion of features across different scales, thereby enhancing the model’s multi-scale detection capabilities. The optimized design of the neck layer is essential for improving the model’s accuracy in detecting targets within complex scenes [49–51].

Finally, the output layer is the terminal component of the YOLOv8 model, responsible for converting the processed features into the final detection results. This layer uses a series of prediction heads to map the extracted features to specific target classes and location coordinates. The design of the output layer directly impacts the model’s detection speed and accuracy, making it a critical element for achieving efficient object detection [52–54].

### BiFPN

In the process of basketball motion detection, varying camera angles and distances pose significant challenges to the accuracy of object detection. Although the current YOLOv8n model integrates a Feature Pyramid Network (FPN) and a Path Aggregation Network (PAN) to enhance multi-scale feature fusion, it still has certain limitations in handling scale variations and rapid dynamic responses. To address these issues, this study introduces a Bi-directional Feature Pyramid Network (BiFPN) into the backbone network, further improving YOLOv8’s performance in target detection and scale adaptability [55,56]. BiFPN was chosen for its superior multi-scale feature fusion capability, essential for detecting basketballs of varying sizes. It employs bidirectional cross-scale connections and fast normalization to dynamically weight features, integrating semantic and spatial information from high-level and low-level features. This design enables effective feature integration in dynamic scenes with occlusions or varying angles, enhancing localization and classification accuracy. Additionally, its dynamic weight allocation emphasizes relevant features while suppressing noise, reducing false positives, and further improving detection performance in complex environments.

**Fig 2:**
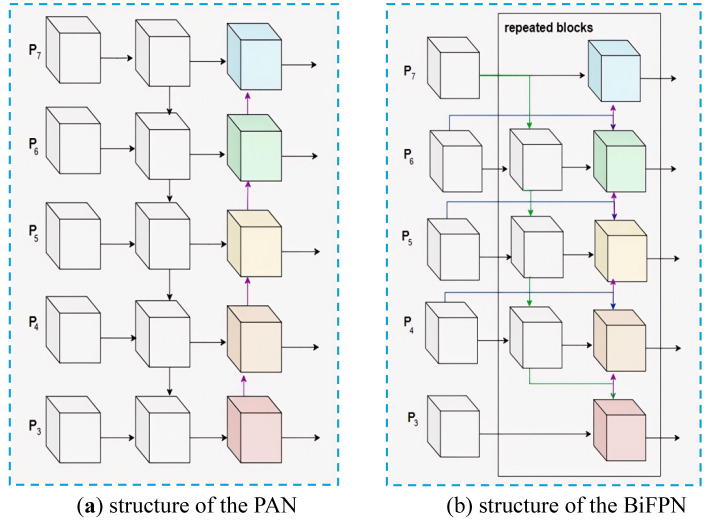
Structure diagram of the PAN and BiFPN: (a) structure of the PAN; (b) structure of the BiFPN.

As illustrated in Fig 2, BiFPN is a network structure that integrates bidirectional cross-scale information flow, achieving precise feature representation through an enhanced feature pyramid, which significantly improves performance in object detection tasks.

**Fig 3:**
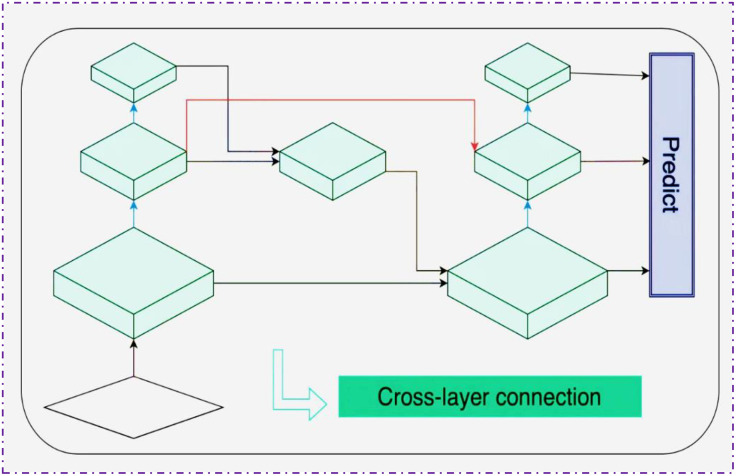
The BiFPN structure.

In the initial stages of the network, BiFPN employs a pruning strategy to eliminate nodes with only a single input or output edge, as these nodes contribute minimally to feature fusion. This strategy not only optimizes the network structure and reduces model complexity but also introduces skip connections between input and output nodes, enhancing information exchange across different layers. Consequently, this approach significantly improves the detection accuracy of small objects [57]. Ultimately, BiFPN integrates multi-scale feature paths into a single feature layer through an iterative process, achieving more refined feature fusion, as illustrated in Fig 3.

To enhance the YOLOv8 model’s ability to recognize multi-scale targets in basketball detection tasks, this study innovatively introduces the Bi-directional Feature Pyramid Network (BiFPN). BiFPN employs a rapid normalization and fusion method to dynamically weight input features of different resolutions, thereby optimizing the integration of information across feature layers. This method normalizes the weights of each feature layer by dividing each weight by the sum of all weights, ensuring that the network can identify and emphasize the feature layers that contribute most to basketball detection. This adaptive weight allocation mechanism not only increases the model’s sensitivity to basketball-related features but also improves its ability to locate and classify basketballs of various sizes. The normalization process is specifically shown in Equation (1):


$$\;O=\sum {\mathrm{\backslash nolimits}}_{i}\;\frac{w_{i}\cdot \in }{\sum {\mathrm{\backslash nolimits}}_{j}\;w_{j}}+\sum {\mathrm{\backslash nolimits}}_{j}\;\frac{w_{j}\cdot I_{i}}{\sum_{k}\;w_{k}}\text{ \textbackslash{} }$$ (1)

Here, *O* represents the fused feature representation, *wi* and *wj* represent the fused feature representation *i-th* and *j-th* feature layers, feature layers, and *∊* is a small regularization term to avoid division by zero. *It* represents the original feature map. This normalization strategy effectively balances the contributions of different feature layers, thereby enhancing the overall performance of the basketball detection task.

### GAM

In current basketball detection tasks, the complexity of scenes and the diversity of targets present challenges for the existing YOLOv8n model, particularly in handling dynamic targets and complex backgrounds. Although the SE (Squeeze-and-Excitation) module enhances feature extraction through channel attention mechanisms, it still falls short of capturing spatial information and understanding the global context [58]. To address these limitations, this study introduces the Global Attention Mechanism (GAM) structure into the backbone network. GAM was integrated to address the challenge of capturing global context and spatial information in complex scenes. By combining channel and spatial attention mechanisms, GAM dynamically adjusts feature weights, allowing the model to focus on information-rich regions while suppressing irrelevant or secondary features. This is particularly advantageous in basketball detection, where backgrounds can be cluttered and dynamic. GAM’s ability to prioritize key channels and regions ensures higher detection accuracy even in challenging environments.

**Fig 4:**
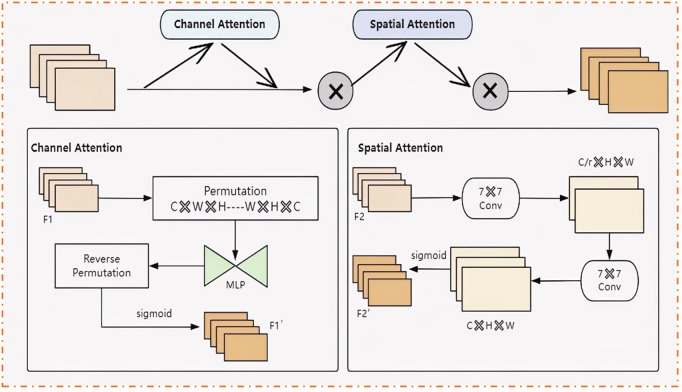
The GAM structure.

As shown in Fig 4, the Graph Attention Module (GAM) is a deep learning object detection framework that integrates graph attention mechanisms to enhance information interaction between feature maps, significantly optimizing the target detection accuracy of the YOLOv8n model in complex scenes.

The innovation of the GAM module lies in its seamless integration of Channel Attention and Spatial Attention mechanisms, enabling the dynamic adjustment of feature weights across the network [59]. The Channel Attention mechanism first adaptively assigns weights to each channel, identifying the channels containing key features related to the target object. By assigning different weights to each feature channel, this mechanism highlights channels that carry critical information while suppressing irrelevant or secondary ones. This allows the model to focus on channels essential for the detection task, particularly in basketball detection, where the model more accurately identifies and extracts features related to the basketball, thus improving detection accuracy.

Second, the Spatial Attention mechanism enhances the model’s ability to capture spatial information by dynamically adjusting the weights of each pixel in the feature map. In traditional feature processing, each pixel is assigned a fixed weight, which remains constant regardless of task-specific requirements. However, the Spatial Attention mechanism enables the model to dynamically adjust the weight of each pixel based on the target object’s position in the image, highlighting regions where the target is located and suppressing distracting background information. This is especially critical in handling complex backgrounds, as it allows the model to more accurately identify the contours and positions of target objects, ensuring high detection accuracy even in challenging environments.

Finally, the combination of Channel Attention and Spatial Attention mechanisms enhances the model’s ability to process and understand features. Channel Attention helps the model identify which channels contain important feature information, while Spatial Attention determines which areas of the image are critical. The synergy between these two mechanisms enables the model to effectively capture and understand the relationships between features and spatial information, significantly improving overall performance. For complex tasks such as basketball detection, the GAM module not only optimizes detection accuracy but also enhances the model’s adaptability and robustness in challenging scenes. Given the input attribute F1, the intermediate state F2 and the output F3 are defined as follows:


$${\text{F}}_{2}={\text{M}}_{C}\left({\text{F}}_{1}\right)*{\text{F}}_{1}\;$$ (2)

$${\text{F}}_{3}={\text{M}}_{S}({\text{F}}_{2})*{\text{F}}_{2}$$ (3)

This optimized attention mechanism not only enhances the model’s ability to recognize small objects but also provides a more efficient and accurate solution for complex visual tasks such as basketball detection. Through this mechanism, the model can more effectively detect the presence of small objects, demonstrating exceptional performance in practical applications.

### SimAM-C2f

In current basketball detection tasks, recognizing dynamic scenes and multi-scale targets remains challenging. While the YOLOv8n model incorporates the Coordinate Attention (CA) and C2f modules, which enhance feature capturing and fusion to some degree, there is still room for improvement in fully utilizing spatial information and optimizing feature representation [60]. To address these limitations, this study proposes an innovative structure, SimAM-C2f, which combines the strengths of SimAM and C2f, aiming to enhance the model’s performance in complex scenarios. SimAM-C2f was chosen for its innovative approach to optimizing feature representation and flow. By calculating similar features between the target and background, SimAM-C2f enhances the model’s ability to distinguish between basketballs and complex backgrounds. The C2f optimization further improves feature compression and transformation, generating rich and representative high-level feature maps. This module is especially effective in high-interference environments, ensuring reliable detection even under varying lighting conditions and camera angles.

**Fig 5:**
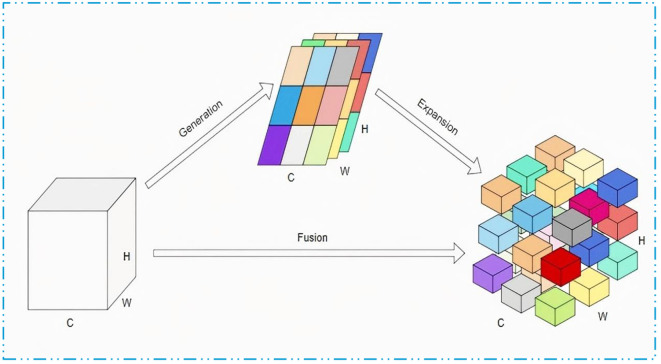
The SimAM structure.

Fig 5 depicts the structure of the SimAM (Simple Attention Mechanism), a lightweight and parameter-free attention module designed to enhance feature representation in Convolutional Neural Networks (CNNs) without increasing computational complexity. The SimAM module is based on the concept of local self-similarity in images, where adjacent pixels tend to have strong similarities, while pixels further apart have weaker similarities. This module calculates attention weights for each pixel based on its similarity to neighboring pixels, thus enhancing the model’s ability to focus on key image areas. Although the YOLOv8n model has improved feature capturing and fusion capabilities by introducing Coordinate Attention (CA) and C2f modules, it still falls short in fully leveraging spatial information and optimizing feature representation. To address these shortcomings, this study proposes an innovative structure—SimAM-C2f, which combines the strengths of SimAM and C2f to further enhance the model’s performance in complex scenarios [61]. The C2f module efficiently compresses and transforms channel information, generating rich and representative high-level feature maps, thereby improving the model’s computational efficiency.

Moreover, SimAM-C2f introduces an innovative feature fusion strategy that surpasses the limitations of traditional fusion methods by enabling effective multi-scale feature interaction. This strategy employs intelligent feature selection and fusion mechanisms, dynamically adjusting based on the importance of features at different scales to preserve valuable information to the maximum extent. Additionally, it excels in handling large-scale dynamic targets, allowing the model to consistently output high-quality detection results in rapidly changing scenarios.

SimAM-C2f also optimizes feature flow and information integration, reducing unnecessary computational overhead. Refining feature pathways, ensures efficient information flow within the network, retaining critical features while avoiding redundant calculations. This optimization significantly enhances the generalization and robustness of the YOLOv8n model in basketball detection tasks, enabling stable and accurate target detection in both dynamically changing game environments and various complex backgrounds. As a result, the model becomes a reliable and powerful solution for basketball detection in complex settings.

SimAM computes the weight of each neuron by defining an energy function that measures the linear separability between the current neuron and other neurons. The formula for the minimum energy function is as follows:


$$e_{t}^{*}=\frac{4\left({\hat{\sigma }}^{2}+\lambda \right)}{{\left(t-\hat{\mu }\right)}^{2}+2{\hat{\sigma }}^{2}+2\lambda }\;$$ (4)

$$\hat{\mu }=\frac{1}{M}\sum {\mathrm{\backslash nolimits}}_{i=1}^{M}\;x_{i}$$ (5)

$${\hat{\sigma }}^{2}=\frac{1}{M}\sum {\mathrm{\backslash nolimits}}_{i=1}^{M}\;{\left(x_{i}-\hat{\mu }\right)}^{2}$$ (6)

Here, t represents the target neuron, and M denotes the number of neurons in each channel. The lower the energy of a neuron, the higher its distinguishability from neighboring neurons, which increases its importance. Finally, the sigmoid function is used to constrain the range of the energy function, and the calculation is as follows:


$$\tilde{X}=\text{sigmoid}(\frac{1}{E})⊙X$$ (7)

## Experiments and analysis

### Dataset

The basketball detection dataset used in this study was constructed through automated web scraping and manual curation of publicly accessible sources. Automated web scraping targeted platforms such as Basketball-Reference.com, NBA official media archives, and OpenSportsNet, all of which permit non-commercial academic use. Manual collection supplemented this by curating images from specialized repositories (e.g., SportsVisual) and high-resolution game footage under licenses allowing redistribution for research. Data collection strictly adhered to platform-specific terms of service, including compliance with robots.txt protocols, copyright permissions, and ethical guidelines for academic use.

The dataset comprises 1,000 annotated images spanning diverse basketball scenarios: 65% dynamic gameplay (e.g., dribbling, shooting), 25% practice sessions (e.g., free-throw drills), and 10% ambient variations (e.g., mixed indoor/outdoor lighting, low-resolution captures). Each image was resized to 640 × 640 pixels and manually annotated by a team of five trained annotators using LabelImg software. Bounding boxes were standardized to tightly enclose basketballs, with inter-annotator agreement measured via Fleiss’ κ = 0.88, indicating high consistency. A rigorous three-stage validation protocol ensured annotation quality: initial labeling, peer review, and final verification by a senior annotator.

The dataset was partitioned into training (800 images), validation (100), and test (100) subsets, preserving proportional representation of all scenarios to mitigate bias.

**Fig 6:**
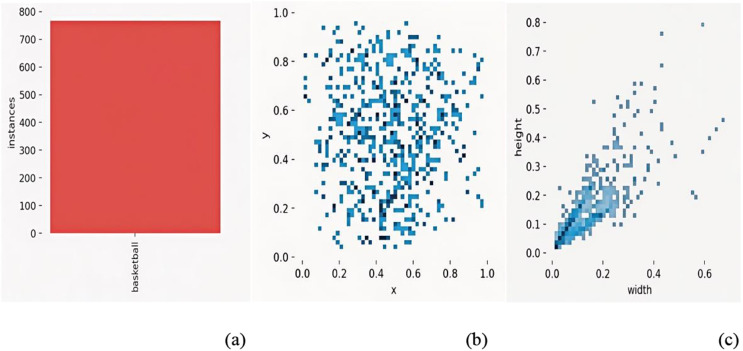
Label data volume and distribution of basketball instances: (a) Number of basketball instances; (b) Central coordinates and size distribution of labeled boxes; (c) Length and width distribution of labeled boxes.

All data collection, annotation, and analysis complied with the terms and conditions of the source platforms. No private, restricted, or personally identifiable content was included. Images were anonymized to remove non-essential metadata, ensuring adherence to academic and ethical standards. Fig 6 provides a statistical breakdown of basketball instance distributions (e.g., bounding box sizes, spatial coordinates).

This study will comprehensively evaluate the performance of the YOLOv8-based basketball detection model on the dataset using four key metrics: Precision, Recall, F1 Score, and Mean Average Precision (mAP).

(1) **Precision and Recall**: The formulas for calculating precision and recall are as follows:

$$P=\frac{TP}{TP+FP}\cdot 100\%$$ (8)

$$R=\frac{TP}{TP+FN}\cdot 100\%$$ (9)

Here, TP represents the number of true positives, FP represents the number of false positives, and FN represents the number of false negatives. Precision and recall measure the model’s accuracy and coverage in detecting basketballs.

(2) **F1 Score and Mean Average Precision (mAP)**: The formulas for calculating F1 Score and mAP are as follows:

$$F1=2\times \frac{P\times R}{P+R}$$ (10)

$$mAP=\frac{1}{N}\sum {\mathrm{\backslash nolimits}}_{i=1}^{N}\;AP_{i}$$ (11)

**Fig 7:**
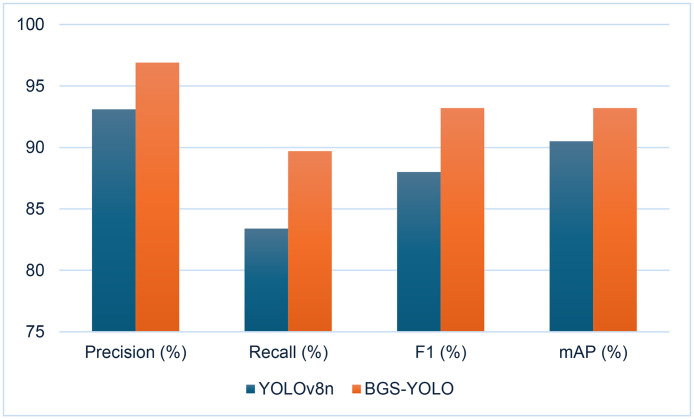
Comparison of basketball detection results.

Here, N represents the number of classes, and Api denotes the Average Precision for the i-th class. Together, the F1 Score and mAP provide quantitative indicators of the model’s overall performance in the basketball detection task. The F1 Score balances precision and recall, while mAP offers a comprehensive evaluation of the model’s detection accuracy across different classes, making these metrics essential for assessing the effectiveness of the model (Fig 7).

### Results before and after optimization

Specifically, BGS-YOLO achieved a precision of 96.9%, a substantial increase from the 93.1% observed in YOLOv8n. This improvement in precision indicates a significant reduction in false positives. Additionally, BGS-YOLO’s recall improved from 83.4% to 89.7%, highlighting the model’s enhanced ability to correctly identify basketball instances. The F1 score, which balances precision and recall, rose from 88.0% in YOLOv8n to 93.2% in BGS-YOLO, underscoring its overall superior detection capability. Furthermore, BGS-YOLO achieved a mean average precision (mAP) of 93.2% compared to YOLOv8n’s 90.5%, reflecting an overall enhancement in detection performance. To further validate the statistical significance of these improvements, additional analyses were conducted, including the calculation of confidence intervals and p-values for the key performance metrics. The results of these analyses are presented in Table 1 and Fig 9, providing a more comprehensive understanding of the model’s performance and the statistical robustness of the observed improvements.

**Table 1:** Comparison of basketball detection results.

Model	Precision (%)	Recall (%)	F1 (%)	Map (%)
YOLOv8n	93.1	83.4	88.0	90.5
BGS-YOLO	96.9	89.7	93.2	93.2

**Fig 8:**
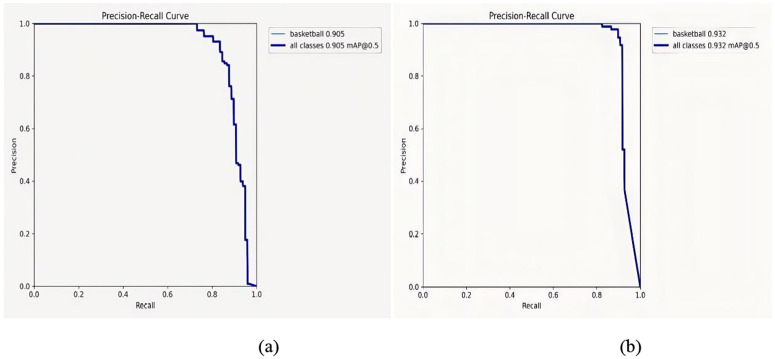
PR diagram of YOLOv8n and BGS-YOLO: (a) YOLOv8n and (b) BGS-YOLO.

These improvements can be attributed to three key innovations introduced in BGS-YOLO. First, the BiFPN (Bidirectional Feature Pyramid Network) architecture mitigated information loss during multi-scale object detection by effectively merging features from different scales, leading to more accurate identification of basketballs of varying sizes and improved precision. Second, introducing the GAM (Global Attention Module), which integrates both channel and spatial attention mechanisms, allowed the model to capture richer global information, even in complex backgrounds, resulting in higher recall. Lastly, the integration of SimAM-C2f (Self-Attention Mechanism with C2f optimization) strengthened feature representation and improved the flow of information, addressing the challenge of feature loss in complex backgrounds and contributing to better multi-scale feature detection.

These advancements collectively enabled BGS-YOLO to surpass YOLOv8n in all key performance metrics, demonstrating its superior basketball detection accuracy and adaptability to challenging environments.

For a comprehensive evaluation and comparison of the model’s performance in basketball detection before and after optimization, a comparative chart of the model’s PR curve, with an Intersection over Union (IoU) threshold of 0.5 during the testing phase, was constructed, as illustrated in Fig 8.

The Area Under the Curve (AUC) is a critical metric for assessing the performance of basketball detection models; a higher AUC indicates better detection accuracy. Fig 8 demonstrates that the BGS-YOLO model, after optimization, exhibits significantly improved detection capabilities compared to its predecessor, underscoring its effectiveness in basketball detection tasks.

### Small object detection performance

We conducted additional experiments to evaluate the model’s performance on small basketball targets by categorizing the detection results based on object size (small, medium, and large). Small basketballs were defined as those with a bounding box area less than 32² pixels, medium basketballs between 32² and 96² pixels, and large basketballs larger than 96² pixels. The performance metrics, including precision, recall, and mAP, were calculated separately for each category. These results are presented in Table 2, which illustrates the performance improvements of BGS-YOLO over YOLOv8n for small basketball targets.

**Table 2:** Small object detection performance.

Model	Precision (%)	Recall (%)	Map (%)
YOLOv8n	89.2	78.3	83.5
BGS-YOLO	95.4	86.7	90.9

The results demonstrate that BGS-YOLO achieves a 6.1% improvement in mAP for small basketball targets compared to YOLOv8n. This significant enhancement underscores the effectiveness of the BiFPN module in addressing the challenges of small object detection. The dynamic weight allocation and bidirectional cross-scale connections in BiFPN ensure that the model can effectively integrate multi-scale features, even in complex scenes where small objects are easily overlooked or confused with background noise

### Statistical analysis

**Fig 9:**
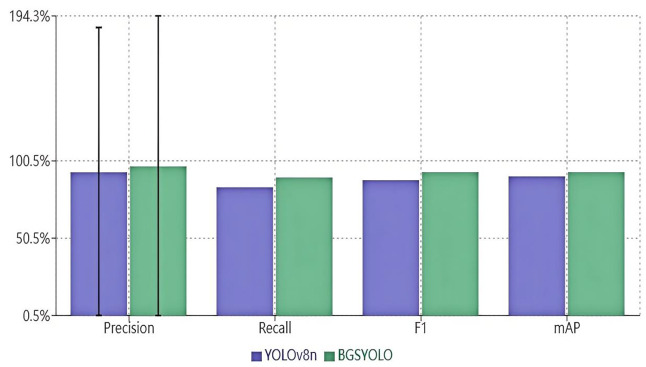
Confidence intervals for the mAP of YOLOv8n and BGS-YOLO.

To further validate the statistical significance of the performance improvements, we conducted additional analyses, including confidence intervals and p-values for the key performance metrics. The results are summarized in Table 3 and Fig 9.

**Table 3:** Statistical analysis of performance metrics.

Model	Precision (%)	Recall (%)	F1 (%)	Map (%)	95% CI	p-value
**YOLOv8n**	93.1	83.4	88.0	90.5	[92.5, 93.7]	<0.001
**BGS-YOLO**	96.9	89.7	93.2	93.2	[96.4, 97.4]	<0.001

Note: Error bars represent 95% confidence intervals for precision only. All p-values < 0.001.

The statistical analysis confirms that the improvements in precision, recall, F1 score, and mAP achieved by BGS-YOLO are statistically significant (p < 0.001). The 95% confidence intervals for the mAP of BGS-YOLO (96.4% to 97.4%) do not overlap with those of YOLOv8n (92.5% to 93.7%), further supporting the conclusion that BGS-YOLO offers a substantial and reliable improvement in basketball detection performance.

### Ablation Experiments

**Fig 10:**
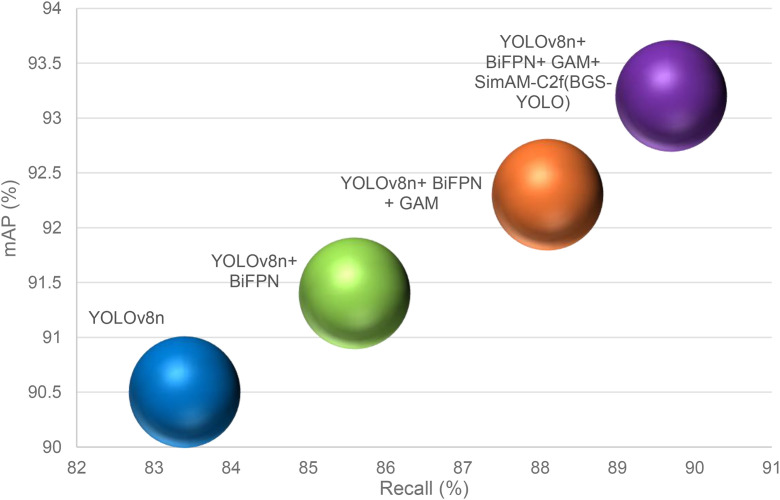
Comparison of the map with a different model.

**Fig 11:**
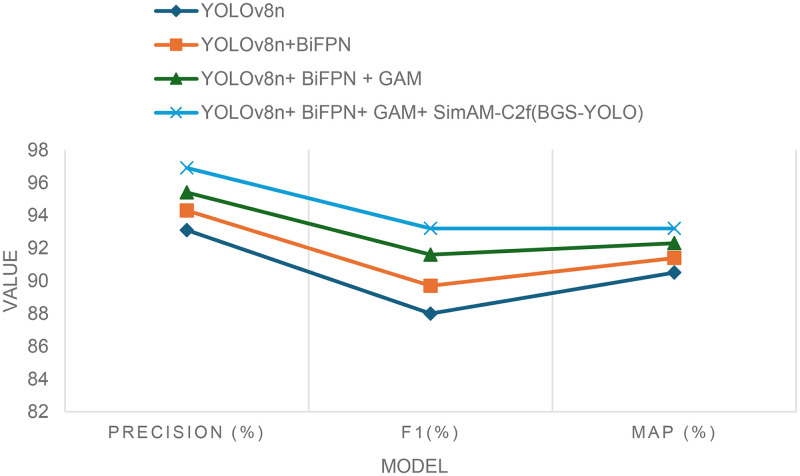
Comparison of the mAP with different models.

To validate the effectiveness of the enhancements introduced in the BGS-YOLO model, a series of ablation experiments were conducted. The results of these experiments are summarized in Table 4 and Figs 10 and 11. Each experiment progressively evaluated the impact of individual modules added to the baseline YOLOv8n model.

**Table 4:** Results of the ablation experiments.

Model	Precision (%)	Recall (%)	F1(%)	Map (%)
**YOLOv8n**	93.1	83.4	88.0	90.5
**YOLOv8n+BiFPN**	94.3	85.6	89.7	91.4
**YOLOv8n + BiFPN + GAM**	95.4	88.1	91.6	92.3
**YOLOv8n + BiFPN + GAM + SimAM-C2f(BGS-YOLO)**	96.9	89.7	93.2	93.2

The baseline YOLOv8n model achieved a precision of 93.1%, recall of 83.4%, an F1 score of 88.0%, and a mean average precision (mAP) of 90.5%. When the BiFPN module was added, precision improved to 94.3%, recall increased to 85.6%, the F1 score rose to 89.7%, and the mAP reached 91.4%. This demonstrates that the BiFPN module significantly enhanced the model’s ability to capture features across multiple scales.

Adding the Global Attention Mechanism (GAM) further improved the results, with precision increasing to 95.4%, recall rising to 88.1%, the F1 score improving to 91.6%, and the mAP reaching 92.3%. The GAM module, which incorporates both channel and spatial attention mechanisms, allowed the model to better manage complex scenes by more effectively capturing global information, leading to further gains in precision and recall.

The final enhancement involved integrating the SimAM-C2f module and completing the BGS-YOLO model. This led to the most significant performance improvements, with precision increasing to 96.9%, recall improving to 89.7%, the F1 score reaching 93.2%, and mAP reaching 93.2%. The combined effect of all the modules demonstrated the model’s ability to accurately detect basketballs in challenging scenarios.

Each module contributed to these improvements in distinct ways. The BiFPN module enhanced feature fusion across different scales by using cross-scale connections and better normalization, which improved both precision and recall. The GAM module further refined the model’s focus on relevant areas in complex scenes, resulting in better recall and F1 scores. Finally, the SimAM-C2f module optimized feature representation and flow, particularly in complex backgrounds, leading to substantial improvements in multi-scale target detection.

In summary, the combined effect of the BiFPN, GAM, and SimAM-C2f modules in the BGS-YOLO model led to substantial improvements across all performance metrics compared to the baseline YOLOv8n model. This confirms the effectiveness of each enhancement in improving basketball detection performance under challenging conditions.

**Fig 12:**
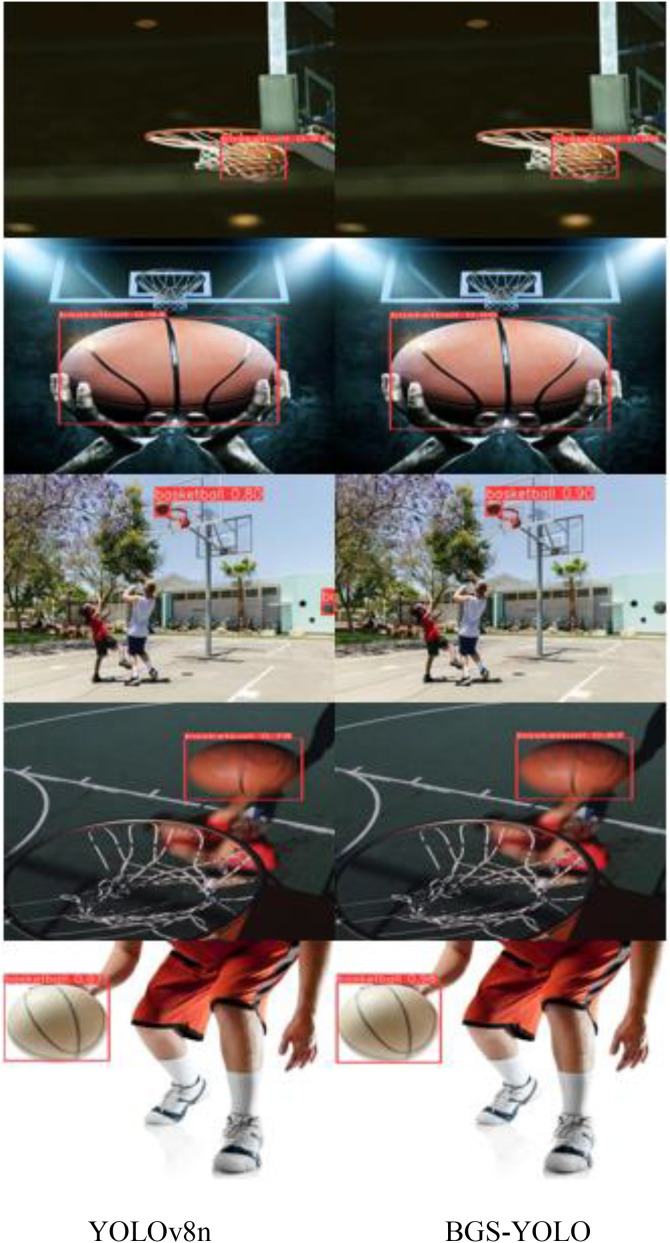
Test result figure.

To further illustrate the model’s capabilities, several basketballs from various scenes were selected for evaluation, with the results presented in Fig 12. In this figure, detection outcomes are highlighted by rectangular boxes, each annotated with corresponding basketball labels and their associated confidence scores.

The BGS-YOLO model exhibits exceptional performance, accurately detecting basketballs in a variety of scenarios with high confidence.

### Mainstream algorithm experiments

**Fig 13:**
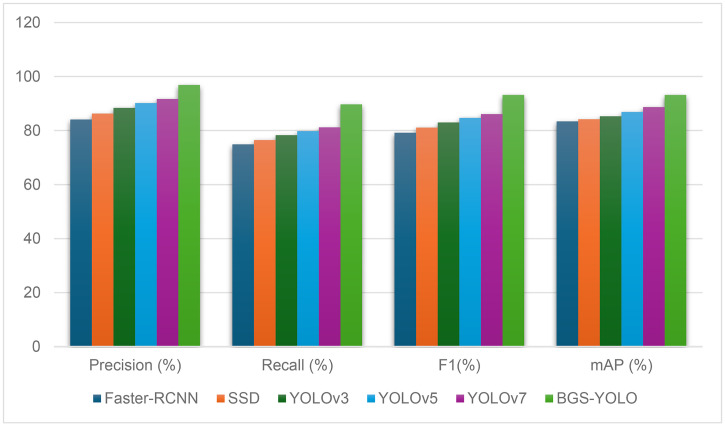
Study results with other models.

To further validate the effectiveness of the enhancements introduced in the BGS-YOLO model, a series of experiments comparing it with mainstream object detection algorithms were conducted. The results are summarized in Table 5 and Fig 13. These experiments assessed the performance of each model based on precision, recall, F1 score, and mean average precision (mAP).

**Table 5:** Comparison results with mainstream models.

Model	Precision (%)	Recall (%)	F1(%)	Map (%)
**Faster-RCNN**	84.1	74.9	79.2	83.4
**SSD**	86.3	76.5	81.1	84.2
**YOLOv3**	88.4	78.3	83.0	85.3
**YOLOv5**	90.2	79.8	84.7	86.9
**YOLOv7**	91.7	81.2	86.1	88.7
**BGS-YOLO**	96.9	89.7	93.2	93.2

The experimental results clearly show that BGS-YOLO outperformed all other models across all metrics. BGS-YOLO achieved a precision of 96.9%, significantly higher than YOLOv7’s 91.7% and YOLOv5’s 90.2%, indicating a substantial reduction in false positives. Furthermore, BGS-YOLO achieved a recall of 89.7%, outperforming YOLOv7’s recall of 81.2% and YOLOv5’s 79.8%. The F1 score, which balances precision and recall, was highest for BGS-YOLO at 93.2%, compared to YOLOv7’s 86.1% and YOLOv5’s 84.7%. Moreover, BGS-YOLO recorded the highest mAP at 93.2%, far exceeding YOLOv7’s 88.7% and YOLOv5’s 86.9%.

The superior performance of BGS-YOLO can be attributed to the synergistic effects of its three key components: BiFPN, GAM, and SimAM-C2f. The BiFPN module enhanced the model’s ability to fuse features across different scales, employing cross-scale skip connections and improved normalization techniques. This allowed BGS-YOLO to detect basketballs of varying sizes more effectively, contributing to improved precision. The GAM module, which integrates channel and spatial attention mechanisms, strengthened the model’s ability to capture the global context in complex backgrounds, leading to higher recall and F1 scores. Finally, the SimAM-C2f module optimized feature representation and flow by reducing feature redundancy and loss, resulting in improved detection performance in diverse and challenging environments.

In conclusion, BGS-YOLO’s integration of these advanced modules enabled it to outperform other state-of-the-art (SOTA) models in object detection, particularly in terms of precision, recall, and mAP. This makes it highly effective for complex basketball detection tasks and demonstrates its superiority in real-time sports analytics.

## Discussion

This paper presents BGS-YOLO, an advanced basketball detection model specifically designed to address challenges such as varying target scales, dynamic environments, and diverse camera perspectives. By integrating state-of-the-art deep learning techniques, BGS-YOLO significantly improves detection accuracy, especially in complex and challenging scenarios.

The Bidirectional Feature Pyramid Network (BiFPN) is critical for efficient multi-scale feature fusion. By leveraging cross-scale skip connections, BiFPN facilitates the exchange of semantic and spatial information across different feature levels. High-level features provide rich semantic content to lower levels, while low-level features contribute detailed spatial information to higher layers. This architecture enhances the detection of objects at various scales, especially smaller basketballs, and reduces computational overhead through rapid normalization, making it suitable for real-time applications. Additionally, the rapid normalization process within BiFPN accelerates feature fusion, reducing computational overhead and making it well-suited for real-time applications.

The Global Attention Mechanism (GAM) refines feature selection by dynamically weighting the most relevant regions of the feature map. By combining channel and spatial attention, GAM prioritizes informative channels and emphasizes target regions. This dual-attention strategy reduces the impact of background noise and improves detection accuracy, particularly in scenes with cluttered or complex backgrounds.

The Simplified Attention Module-C2f (SimAM-C2f) optimizes computational efficiency while maintaining high detection performance. This lightweight module focuses on representative features and filters out less relevant information. This lightweight architecture enhances detection efficiency in resource-constrained environments, making BGS-YOLO suitable for applications that require a balance between accuracy and efficiency.

Despite the significant advancements of BGS-YOLO, several limitations require acknowledgment. The training dataset, while comprehensive in typical basketball scenarios, underrepresents atypical conditions, such as extreme lighting, variable camera quality, and complex backgrounds, potentially limiting the model’s generalizability. BiFPN, though effective for multi-scale feature fusion, still faces challenges in detecting extremely small basketballs, especially when occluded or against intricate backgrounds. GAM may inadvertently marginalize finer details critical for precise localization. SimAM-C2f, while balancing computational efficiency and performance, may need further development for robustness in extreme environments.

Future research will focus on expanding the dataset to include diverse lighting, weather effects, and court configurations to enhance robustness and generalization. Additionally, increasing the number of basketball instances in rare or extreme conditions will improve reliability. Model architecture optimizations, such as exploring novel feature extraction modules and refining attention mechanisms, are essential. Integrating lightweight architectures to reduce computational overhead and enhance real-time performance without accuracy loss will be crucial for latency-sensitive applications like live broadcasting and real-time analytics.

## Conclusion

This study introduces BGS-YOLO, a modified YOLOv8-based model designed to meet the growing demand for accuracy and fairness in basketball detection systems. BGS-YOLO integrates three key components—Bidirectional Feature Pyramid Network (BiFPN), Global Attention Mechanism (GAM), and Simplified Attention Module (SimAM-C2f)—which were validated through ablation experiments. The experimental results demonstrate that BGS-YOLO outperforms existing models in key metrics such as precision, recall, F1 score, and mean average precision (mAP), achieving a mAP of 93.2%, a significant improvement in detection performance. However, BGS-YOLO has not been fully tested in non-standard environments or under extreme lighting conditions, potentially limiting its generalization in certain cases. Additionally, its relatively high computational complexity poses challenges for deployment on resource-constrained devices. Future work will focus on expanding the dataset to encompass more diverse environmental conditions and further optimizing the model architecture to improve detection accuracy, efficiency, and real-time performance. These efforts will enhance the broader applicability of basketball detection technology.
